# Anti-Inflammatory and Cytotoxicity Effects of *Cudrania tricuspidata* Fruits Vinegar in a Co-Culture System with RAW264.7 Macrophages and 3T3-L1 Adipocytes

**DOI:** 10.3390/foods9091232

**Published:** 2020-09-04

**Authors:** Jun-Hui Choi, Se-Eun Park, Soo-Hwan Yeo, Seung Kim

**Affiliations:** 1 Department of Food Science and Biotechnology, Gwangju University, Gwangju 503-703, Korea; sekai0572@naver.com (J.-H.C.); esca20@naver.com (S.-E.P.); 2 Department of Agro-food Resource, National Academy of Agricultural Science, RDA, Suwon 441-853, Korea; yeobio@korea.kr

**Keywords:** vinegar, coculture techniques, macrophages, adipocytes, inflammation

## Abstract

Vinegar has been found to have in vitro improvement effect on inflammatory biomarkers, and clinically used to improve inflammation and obesity-related diseases. This study was designed to analyze in vitro anti-inflammatory effects of *Cudrania tricuspidata* fruits vinegar (CTFV) in a co-culture system with macrophages and adipocytes. We analyzed the physicochemical properties and polyphenolic ingredients of CTFV, and investigated in vitro anti-inflammatory effects of CTFV in a co-culture system with macrophages and adipocytes. The cells were cultured in the presence of CTFV for 24 h in contact with each other, then, harvested. The levels of monocyte chemoattractant protein (MCP)-1, tumour necrosis factor (TNF)-α, inducible nitric oxide synthase (iNOS), nitric oxide (NO), and interleukin (IL)-6 were evaluated by using the Griess reagent, western blot, or enzyme-linked immunosorbent assay assays. We found that increasing levels for NO, iNOS, TNF-α, IL-6 and MCP-1 were caused by LPS treatment and co-culture using the contact method, whereas CTFV efficaciously attenuated inflammatory response by improving inflammatory parameters including NO, iNOS, TNF-α, IL-6 and MCP-1. The present study indicates that CTFV might provide a nutraceutical product or functional food resource for improving inflammation processed via the interaction of adipocytes and macrophages.

## 1. Introduction

*Cudrania tricuspidata* is commonly used as food ingredients and a medicinal plant, and its fruit is consumed fresh and dried or used in the preparation of fruit jams and alcoholic drinks. This plant is mostly distributed in East Asia and its fruit is rich in bioactive substances, such as phenolic compounds, flavones, and isoflavonoids [1]. Several studies on the anti-inflammatory effects of this plant and its active ingredients have been reported [2,3,4,5].

The adipose tissue is an active endocrine organ containing diverse cells, including macrophages, adipocytes, and immune and endothelial cells, that release adipokines such as adipose hormones and cytokines, and regulate the levels of adipokines under various conditions [6]. In obesity, the chronic inflammatory response contributes to increased macrophage infiltration, which in turn leads to decreased adiponectin secretion and elevated levels of pro-inflammatory cytokines [7]. The paracrine interactions between adipocytes and macrophages in adipose tissues are activated by polysaccharides, and fatty acids, and are involved in the regulation of the adipokine profile [7,8]. To improve chronic inflammation in obesity, investigation of the paracrine interactions between macrophages and adipocytes is crucial. We therefore chose two cell lines, RAW264.7 macrophages and 3T3-L1 adipocytes, for analysis of the paracrine interactions using a co-culture system. RAW264.7 macrophage, a monocyte cell line derived from BALB/c mice, plays an important role in the immune response involving phagocytosis, antigen presentation, and immune regulation to release various cytokines and growth factors. 3T3-L1 adipocytes derived from mouse 3T3 cells induce the accumulation and synthesis of triglycerides. The cells are sensitive to lipolytic and lipogenic hormones, and release pro-inflammatory cytokines, including monocyte chemoattractant protein 1 (MCP-1) and tumor necrosis factor-α (TNF-α). The two cell lines can produce an inflammatory response and are involved in the regulation of cytokines and hormones related to immune activity and growth.

Previous studies have shown that vinegar treatment decreased the levels of inflammatory cytokines [9,10], and biomarkers including mitogen-activated protein kinases (MAPKs), cyclooxygenase (COX)-2, inducible nitric oxide synthase (iNOS), and nitric oxide (NO) [9,10,11]. Vinegar has been found to possess inhibitory effects against immunoglobulin E (IgE) production, immune cell infiltration, Th1 or Th17 responses, and Toll-like receptor (TLR)4-induced inflammatory response [10,11,12]. Moreover, vinegar supplementation reduced the levels of interleukin-6 (IL-6), an inflammatory cytokine, and the biomarkers nitric oxide (NO) and TNF-α in a clinical study [13,14,15,16]. Recently, vinegars have been reported to reduce body fat and glucose levels, as well as exert anti-oxidant, anti-tumor, and anti-microbial effects [15,16,17,18,19]. The biological activities of fruit vinegars have been studied to exploit the beneficial effects of vinegar. Some plant fruits, including *Schizandra chinensis*, black raspberries, apples, blueberries, and *Vitis coignetiae*, have been used to produce fruit vinegar [20,21,22]. To date, there is no information on the in vitro anti-inflammatory efficacy of *C. tricuspidata* fruit vinegar (CTFV) in a co-culture system with macrophages and adipocytes. Therefore, our study was designed to evaluate the in vitro anti-inflammatory effects of CTFV in a co-culture system with macrophages and adipocytes.

## 2. Materials and Methods

### 2.1. Chemicals, Reagents, and Antibodies

Quercetin, rosmarinic acid, coumaric acid, cinnamic acid, taxifolin, ferulic acid, *p*-coumaric acid, rutin, isovanillic acid, chlorogenic acid, protocatechuic acid, gallic acid, *p*-hydroxybenzoic acid, caffeic acid, gastrodin, *p*-hydroxybenzyl alcohol, ethylenediaminetetraacetic acid (EDTA), dimethyl sulfoxide (DMSO), hematoxylin and eosin, and parishin A, B, C, and E were purchased from Sigma-Aldrich (St. Louis, MO, USA). Streptomycin, penicillin, fetal bovine serum (FBS), and Dulbecco’s modified Eagle’s medium (DMEM) were purchased from Invitrogen (Carlsbad, CA, USA). Primary antibodies against iNOS was purchased from Santa Cruz Biotechnology, Inc. (Santa Cruz, CA, USA). Prestained protein ladder (PageRuler TM Plus) was purchased from Thermo Scientific (Rockford, IL, USA). Other reagents were commercially available and of special grade.

### 2.2. Wine and Vinegar Preparation

Fresh *C. tricuspidata* fruits were collected from a farm located in Milyang, Gyeongsangnam-do, Republic of Korea and immediately stored at −20 °C until use. Before preparation of the wine, the frozen fruit was homogenized with tap water at a 1:2 ratio using a multipurpose high-performance hand blender (Lacuzin, China). The sets of mixture were raised to 12 °Brix by adding sugar and sterilized for 90 min at 85 °C. The yeast strain (*Saccharomyces cerevisiae* Fermivin) was cultivated by inoculating into malt medium (12 °Brix) and incubating at 25–27 °C for 5 days under shaking at 120 rpm. The fermentation process was then initiated by inoculating 6 L of the homogenized fruit slurry in a 10 L glass jar with 5% of the yeast culture. The fermentation jar was then incubated at 26 °C for 10 days with manual shaking twice daily. At the end of fermentation, the two wine phases (liquid and solid) were separated by centrifugation at 9000× *g* for 15 min, and the liquid phase was filtered through a 110 mm filter (Whatman filter paper no. 2). The *C. tricuspidata* fruit wine was then sterilized for 30 min at 85 °C. To prepare the vinegar, equal volumes of the *C. tricuspidata* fruit wine and traditional starter vinegar [23] were mixed and incubated at 30 °C for 60 days. During incubation, fresh wine feeding (half of the volume of the vinegar preparation) and acidity measurements were conducted every twelve days.

### 2.3. HPLC Analysis

HPLC was performed using an HPLC system (Waters, Milford, MA, USA) equipped with a Waters 996 DAD with a ZORBAX Eclipse XDB-C18 column (250 mm × 4.6 mm, 5 µm; Agilent Technologies, Inc., Santa Clara, CA, USA), and 2690 separation module as previously described [24]. For analysis of phenolic acids and flavonoids, the mobile phase comprised 0.1% formic acid in 10% acetonitrile of solvent A, and 0.1% formic acid in 90% acetonitrile of solvent B. The mobile phase ratio was maintained at A:B 100:0 for 0–5 min, 100:0 for 5–10 min, 88:12 for 10–40 min, 30:70 for 40–45 min, and 100:0 for 45–50 min at 0.8 mL/min of a flow rate. The quantification of each compounds was analyzed at 280 nm based on peak areas.

The parishin derivatives were analyzed by the previously described method [24]. The mobile phase comprised 0.1% formic acid in ionized water of solvent A, and 0.1% formic acid in methanol of solvent B. The mobile phase ratio was maintained at A:B 95:5 for 0–5 min, 85:15 for 5–10 min, 45:55 for 10–25 min, and 95:10 for 25–40 min at 0.8 mL/min of a flow rate. The quantification of each compounds was analyzed at 220 nm based on peak areas.

Twenty phenolic compound standards were used for calibration curves: quercetin, rosmarinic acid, coumaric acid, cinnamic acid, taxifolin, ferulic acid, *p*-coumaric acid, rutin, isovanillic acid, chlorogenic acid, protocatechuic acid, gallic acid, *p*-hydroxybenzoic acid, caffeic acid, parishin A, B, C, E, *p*-hydroxybenzyl alcohol, and gastrodin by the previously described method [24]. 50, 100, 250, and 500 µg/mL of the standard solutions were dissolved in DMSO. The main compounds of CTFV were identified based on the retention times of the standards. These compounds were spectrophotometrically quantified by using the peak intensities to those of standard curves.

### 2.4. Cell Culture and Co-Culture of Macrophages and Adipocytes

Cell culture was carried out as previously described [25]. 3T3-L1, and Raw264.7 cells were obtained from the American Type Culture Collection (Manassas, VA, USA). Raw264.7 cells were cultured in DMEM added with 100 µg/mL streptomycin, 100 U/mL penicillin, and 10% FBS. The cells were incubated in humidified air (5% CO_2_, 95% air) at 37 °C. The media were changed every 2 days. 3T3-L1 cells were cultured as previously described [26]. In the contact method described previously [26,27], 5.0 × 10^5^ cells/well of serum-starved 3T3-L1 were cultured in a 24-well plate. 2.0 × 10^5^ cells/well of RAW264.7 were placed above the 3T3-L1. The cells were cultured in 2% FFA-free BSA medium for 24 h in the presence of CTFV (0, 50, 100, and 200 µg/mL) in contact with each other, then, harvested. The medium supernatants and cell lysate buffers of differentiated RAW264.7 and 3T3-L1 were used to harvest the co-cultures.

### 2.5. Cell Viability Assay

Using the 3-(4,5-dimethylthiazol-2-yl)-2,5-diphenyltetrazolium bromide (MTT) reduction assay, cell viability was determined to examine possible toxic effects as previously described [25]. Cells were prepared at a density of 1 × 10^4^ cells/well into 96-well plates, and incubated for 24 h before experimental treatments. The sample was dissolved in saline solution. 3T3-L1, and Raw264.7 cells were treated with 10–500 µg/mL CTFV for 24 h. After incubation with MTT for 4 h at 0.5 mg/mL of a final concentration, and 37 °C, the media were removed. To dissolve the formazan crystals, 100 µL of dimethyl sulfoxide was added to each well for 10 min, and absorbance was measured using a microplate reader at 570 nm.

### 2.6. Measurement of IL-6, TNF-α, and MCP-1

Determination of IL-6, TNF-α, and MCP-1 levels in the cells extracts or the culture medium by enzyme-linked immunosorbent assay (ELISA). The IL-6, TNF-α, and MCP-1 levels of the extracts were determined using a commercially available IL-6 or TNF-α ELISA kit (R&D Systems, Minneapolis, MN, USA), and MCP-1 ELISA kit (Sigma-Aldrich, St. Louis, MO, USA). The assays were conducted by the manufacturer’s the instructions provided.

### 2.7. NO Assay

Nitric oxide metabolite from cells was measured by the Griess reaction as previously described [28]. First, each sample of 100 µL was incubated with Griess reagent of 100 µL, containing 0.1% naphthyl ethylenediamine dihydrochloride and 1% sulfanilamide in 2.5% polyphosphoric acid at room temperature (RT) for 10 min. Then, the absorbance was measured at 540 nm with a microplate reader. The concentration of nitric oxide metabolite was determined by measuring the absorbance at 540 and comparing to those of sodium nitrite.

### 2.8. Immunoblotting

Immunoblotting analysis was performed as previously described [25]. The collected cells were washed with phosphate buffer solution (PBS). Then, the harvested cells were lysed by RIPA buffer (Sigma-Aldrich, St. Louis, MO, USA). The concentrations of protein were analyzed by BCA assay (Thermofisher, Waltham, MA, USA). Each extracts of 20 µg were analyzed by 12% sodium dodecyl sulfate-polyacrylamide gel electrophoresis. Then, the proteins were transferred to polyvinylidene difluoride membranes. After blocking in blocking buffer (0.1% Tween 20 [pH 7.5], 150 mM NaCl, and 10 mM Tris-HCl) at RT for 1 h, the membranes were incubated with primary antibodies against beta-actin (1:2500 dilution), and iNOS (1:1000 dilution) at RT for 1 h. The membranes were washed in TBST buffer, and incubated with horseradish peroxidase-conjugated secondary antibodies at RT for 1 h. The membranes were treated with Western Blue Stabilized Substrate for WESTZOL (plus) Western Blot Detection System (Intron Biotechnology, Inc., Seongnam, Korea) to reveal the reaction bands. The signals were detected by a MicroChemi instrument (DNR Bio-imaging Systems, Jerusalem, Israel).

### 2.9. Statistical Analysis

Statistical analysis was performed by the previously described method [5] using SPSS 21 software (SPSS Inc., Chicago, IL, USA). The data collected and analyzed from this study were expressed as mean ± standard deviation (SD). The statistical significance of multiple group comparisons was assessed by one-way analysis of variance followed by a *post-hoc* Tukey’s test. *p*-values less than 0.05 were considered as statistically significant.

## 3. Results

### 3.1. Physicochemical Properties and Polyphenolic Compositions of CTFV

**Figure 1:**
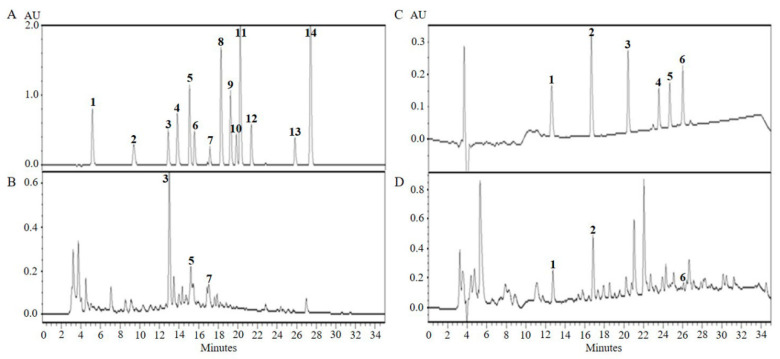
HPLC analysis of polyphenolic ingredients (**B**) and parishin derivatives (**D**) from CTFV. Mixture of authentic standards for polyphenolics (**A**) and parishin derivatives (**C**). *X*-axis is retention time in minutes and *Y*-axis is absorbance unit (AU). 1, gallic acid; 2, protocatechuic acid; 3, chlorogenic acid; 4, *p*-hydroxybenzoic acid; 5, caffeic acid; 6, isovanillic acid; 7, rutin; 8, *p*-coumaric acid; 9, ferulic acid; 10, taxifolin; 11, trans-coumaric acid; 12, rosmarinic acid; 13, quercetin; 14, trans-cinnamic acid (A, left panel). 1, gastrodin; 2, *p*-hydroxybenzyl alcohol; 3, parishin E; 4, parishin B; 5, parishin C; 6, parishin A (C, right panel).

The traditional fermentation process in the described method was used to produce CTFV. After the alcoholic and acetic acid fermentation process, we analyzed various physicochemical properties of CTFV. The pH value of CTFV was found to be 3.4 ± 0.1, and the sugar level was found to be 21.3 ± 0.5 °Brix. The total acidity, the alcohol concentration, and the total organic acid were 11.2 ± 0.3%, 0 ± 0%, and 49.2 ± 0.9 mg/mL. In previous studies [1,24], we found that CTF and its ferment compose various bio-active ingredients such as phenolic, flavonoid, and parishin derivatives. Therefore, we performed HPLC analysis to identify polyphenolic ingredients and parishin derivatives in CTFV (Figure 1). Table 1 and Table 2 shows that the concentrations of chlorogenic acid, caffeic acid, rutin, gastrodin, *p*-hydroxybenzyl alcohol, and parishin A detected in CTFV were 454.1 ± 5.2, 60.8 ± 0.4, 40.1 ± 2.9, 273.1 ± 4.6, 182.9 ± 0.9, and 41.7 ± 0.3, respectively, at each peak compared to standards.

### 3.2. Effect of CTFV on Cell Viability in 3T3-L1, and Raw264.7

**Figure 2:**
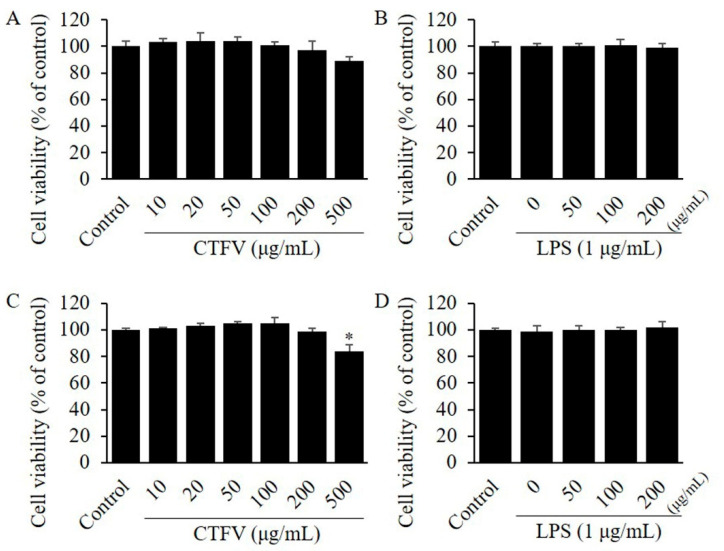
Assessment of cytotoxic effects. MTT assay showing the effect of CTFV on RAW264.7 (**A**,**B**), and 3T3-L1 cells (**C**,**D**) viability. Cells were incubated with the compound at different concentrations (0–500 µg/mL) or LPS at 1 µg/mL for 24 h and cell viability was analyzed by MTT reduction assay. Each value is the mean ± SD of triplicate measurements. * *p* < 0.01, compared with non-treated group. MTT, 3-(4,5-dimethylthiazol-2-yl)-2,5-diphenyltetrazolium bromide; CTFV, *C. tricuspidata* fruits vinegar; LPS, lipopolysaccharide.

The effect of CTFV on cell viability was investigated using two cell lines: 3T3-L1, and Raw264.7 by MTT assay. Each cell line was treated with 30, 50, 100, 200, 300, 500, 700, and 1000 µg/mL CTFV of different concentrations for 48 h. Although CTFV treatment up to 200 µg/mL showed no effects on the viability of non- or LPS-treated Raw264.7 (Figure 2A,B), and non- or LPS-treated 3T3-L1 (Figure 2C,D), 500µg CTFV decreased the viability of 3T3-L1 cell line.

### 3.3. Effects of CTFV on Cytokines Levels in Macrophages or Adipocytes

**Figure 3:**
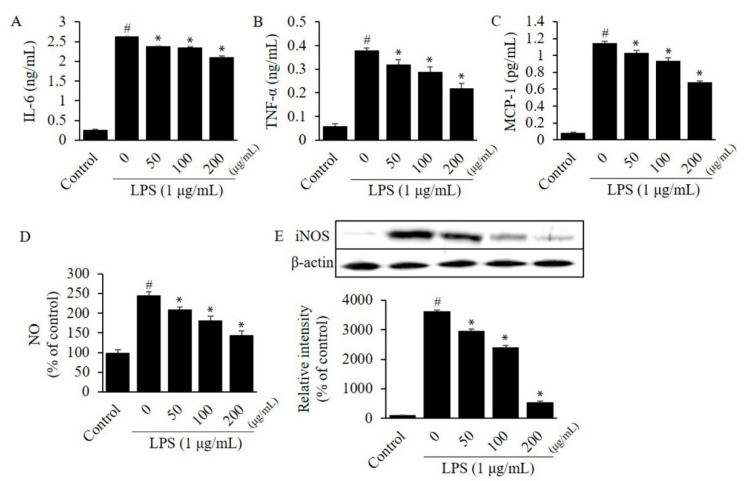
Effect of CTFV on the inflammatory response in RAW264.7 cells. The levels of IL-6 (**A**), TNF-α (**B**), MCP-1 (**C**), NO (**D**), and iNOS (**E**) in RAW264.7 cells were measured by ELISA, the Griess reagent, and western blot analysis. Measurement of these expression or levels in non-treated cells was used as the control group. Each value is the mean ± SD of triplicate measurements. ^#^ *p* < 0.01, compared with non-treated group. * *p* < 0.01, compared with only LPS-treated group. CTFV, *C. tricuspidata* fruits vinegar; IL, interleukin; TNF, tumour necrosis factor; MCP, monocyte chemoattractant protein; NO, nitric oxide; iNOS, inducible nitric oxide synthase; ELISA, enzyme-linked immunosorbent assay; LPS, lipopolysaccharide.

**Figure 4:**
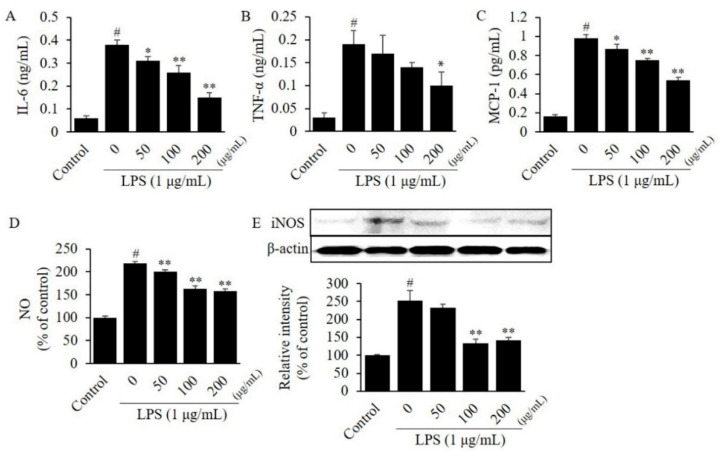
Effect of CTFV on the inflammatory response in 3T3-L1 cells. The levels of IL-6 (**A**), TNF-α (**B**), MCP-1 (**C**), NO (**D**), and iNOS (**E**) in 3T3-L1 cells were measured by ELISA, the Griess reagent, and western blot analysis. Measurement of these expression or levels in non-treated cells was used as the control group. Each value is the mean ± SD of triplicate measurements. ^#^ *p* < 0.01, compared with non-treated group. * *p* < 0.05 and ** *p* < 0.01, compared with only LPS-treated group. CTFV, *C. tricuspidata* fruits vinegar; IL, interleukin; TNF, tumour necrosis factor; MCP, monocyte chemoattractant protein; NO, nitric oxide; iNOS, inducible nitric oxide synthase; ELISA, enzyme-linked immunosorbent assay; LPS, lipopolysaccharide.

To investigate the efficacy of CTFV on the inflammatory response, we evaluated the level of cytokines in LPS-stimulated RAW264.7 or 3T3-L1. The MTT assay revealed that 24 h treatment of RAW264.7 cells with ≤200 µg/mL CTFV resulted in no toxicity, thus, ≤200 µg/mL CTFV was treated in all assays. Protein levels of IL-6, TNF-α, and MCP-1 in a single-cell culture of macrophages RAW264.7 (Figure 3A–C) or adipocytes 3T3-L1 (Figure 4A–C) were remarkably increased by 6.1–14.4-fold, whereas these levels were significantly decreased by CTFV treatment.

### 3.4. Effects of CTFV on Nitrite, and iNOS Levels in Macrophages or Adipocytes

To further investigate the effects of CTFV in macrophage or adipocyte inflammation response, we evaluated the NO metabolite, and iNOS protein expression. Noticeable increase of NO metabolite (2.2–2.5-fold) and iNOS protein levels (2.5–36.3-fold) were observed in RAW264.7 (Figure 3D,E) and 3T3-L1 (Figure 4D,E), compared to non-treated control group. However, CTFV significantly reduced NO metabolite and iNOS expression in both cell lines.

### 3.5. Effects of CTFV on Cytokines Levels in Co-Culture Medium

**Figure 5:**
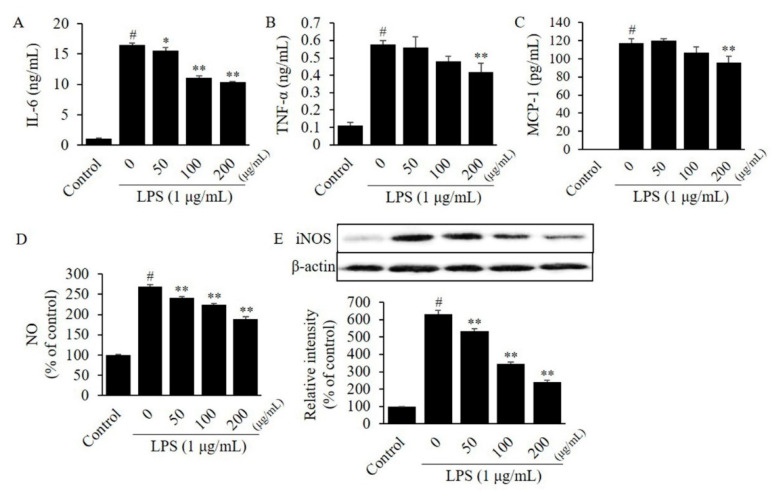
Effect of CTFV on the inflammatory response induced by 24 h co-culture using the contact method. The levels of IL-6 (**A**), TNF-α (**B**), MCP-1 (**C**), NO (**D**), and iNOS (**E**) in the medium co-cultured with RAW264.7 and 3T3-L1 were measured by ELISA, the Griess reagent, and western blot analysis. Measurement of these expression or levels in the non-treated co-culture medium was used as the control group. Each value is the mean ± SD of triplicate measurements. ^#^ *p* < 0.01, compared with non-treated co-culture medium group. * *p* < 0.05 and ** *p* < 0.01, compared with only LPS-treated co-culture medium group. CTFV, *C. tricuspidata* fruits vinegar; IL, interleukin; TNF, tumour necrosis factor; MCP, monocyte chemoattractant protein; NO, nitric oxide; iNOS, inducible nitric oxide synthase; ELISA, enzyme-linked immunosorbent assay; LPS, lipopolysaccharide.

To elucidate the effects of CTFV on an inflammation response in a co-culture system with RAW264.7 and 3T3-L1, we evaluated the level of inflammatory cytokines using the contact method (Figure 5). The levels of IL-6, TNF-α, and MCP-1 in the co-culture medium were increased by 4.35-, 1.38-, and 2.63-fold compared to separate culture of RAW264.7, and by 18.83-, 3.67-, and 2.31-fold compared to separate culture of 3T3-L1. Moreover, IL-6, TNF-α, and MCP-1 levels were significantly higher in LPS-stimulated RAW264.7 or 3T3-L1 than in non-treated control group by 14.6-, 5.3-, and 557.1-fold. CTFV at 200 µg/mL significantly reduced the production of IL-6 (Figure 5A), TNF-α (Figure 5B), and MCP-1 (Figure 5C).

### 3.6. Effects of CTFV on Nitrite, and iNOS Levels in Co-Culture Medium

To further elucidate the effects of CTFV on an inflammation response in a co-culture system with both cell lines, we investigated NO metabolite, and iNOS protein expression using the contact method in co-culture medium (Figure 5). The level of iNOS in the co-culture medium was increased by 4.59-fold compared to separate culture of RAW264.7, and by 2.72-fold compared to separate culture of 3T3-L1. While nitrite and iNOS protein concentrations were increased by 2.7- and 6.3-fold in LPS-stimulated group compared to non-treated control group, treatment with CTFV significantly inhibited the levels of nitrite (Figure 5D) and iNOS protein (Figure 5E) in co-culture medium.

## 4. Discussion

Obesity often causes chronic inflammation, and related studies have revealed that obesity, low-intensity chronic inflammation, and insulin resistance are closely associated with each other [29,30]. Adipose tissues release various adipokines involved in energy homeostasis that interact with macrophages and play a crucial role in the inflammatory response in adipose tissues. Recent studies have focused on new approaches to treat chronic inflammation, obesity, and diabetes by mitigating the inflammation induced by obesity and identifying the relationship between inflammation and adipokines [31,32,33]. In obese conditions, the levels of adipokines secreted from adipocytes increase and cause the infiltration of macrophages into adipocytes, leading to inflammation and increased insulin resistance. Another factor involved in the accumulation of macrophages in the adipose tissues is the management of the dead hypertrophic cells surrounded by adipose tissue macrophages (ATM) [34]. A recent clinical study also reported a link between body mass index (BMI) and the number of macrophages in adipocytes [35]. TNF-α and IL-6 are pro-inflammatory cytokines that are synthesized in white adipose tissues and their concentrations increase with an increase in lipid accumulation [36,37]. MCP-1 is a chemotactic signaling molecule secreted by adipocytes that triggers the recruitment of macrophages [38]. Macrophage infiltration into adipose tissue is closely related to obesity-induced inflammation. Inflammatory intermediaries secreted by adipocytes promote the penetration of immune cells and accelerate macrophage infiltration into the adipose tissues. Stimulated macrophages produce pro-inflammatory cytokines, which upregulate the inflammatory response of obese adipose tissues. The phenotype is shifted from anti-inflammatory M2 macrophages to pro-inflammatory M1 macrophages in obese adipose tissues [39]. The anti-inflammatory M2 macrophages are characterized by the expression of arginase, which blocks the production of anti-inflammatory cytokines and inducible nitric oxide synthase (iNOS) activity, whereas the pro-inflammatory M1 macrophages induce the expression of iNOS, catalyzing the production of NO, and markedly increase the concentrations of pro-inflammatory cytokines such as TNF-α and IL-6 [40]. iNOS expression regulates the infiltration of inflammatory cells and the release of inflammatory cytokines; high levels of iNOS expression disrupt lymphatic endothelial NOS (eNOS) expression through infiltration of macrophages, resulting in decreased immunity [41,42,43].

Vinegar has been used since 300 BC as a food preservative and flavoring agent in Western, European, and Asian countries. While vinegar was once used as an acidic seasoning, its usage has expanded to the medical fields owing to its biological activities, which have been studied extensively. Vinegar contains various bioactive ingredients, including vitamins, minerals, organic acids, carbohydrates, peptides, and polyphenolics, which are beneficial for health [44,45,46]. In particular, fruit vinegars produced from berries, pineapples, grapes, and apples have various polyphenols and flavonoids that are known to possess biological properties such as anti-oxidant and anti-inflammatory effects [10,11,47]. Several studies have investigated the anti-inflammatory effects of fruit vinegars, including fermented fig, *Ficus* spp. (fig), pear, pomegranate, and apple vinegars [47,48,49].

A previous study established the conditions of the fermentation process of CTFV using acetic acid isolated from traditional fermented food; this study primarily focused on the chemical properties of vinegar, the discovery of bacterial strains from traditional foods, free radical scavenging, and sensory evaluation assays [50]. Our study demonstrates the physicochemical properties of CFTV fruit vinegar prepared using *C. tricuspidata* fruits, the detection of polyphenolic ingredients in CTFV, and the anti-inflammatory effects of CTFV in a co-culture system comprising macrophages and adipocytes. We demonstrated that the levels of secreted pro-inflammatory adipokines were elevated in the RAW264.7 and 3T3-L1 cells. Moreover, we showed the efficacy of CTFV in reducing inflammation via paracrine interactions between RAW264.7 and 3T3-L1 cells. We found that the levels of IL-6, MCP-1, NO metabolite, and iNOS were higher in the co-culture medium with RAW264.7 and 3T3-L1 than in those with single cultures (Figure 3, Figure 4 and Figure 5), suggesting that direct cell-to-cell contact centrally causes pro-inflammatory crosstalk between macrophages and adipocytes. The activation of the various factors observed in the co-culture system with RAW264.7 and 3T3-L1 cells was consistent with the increasing levels of NO, iNOS, TNF-α, IL-6, and MCP-1 reported in previous studies [27,51]. Among the factors investigated, we found high expression of MCP-1 in the co-culture compared to that in the single cell culture. In addition, a previous study showed that the increase in MCP-1 during co-culture was primarily due to adipocytes, while that of TNF-α was primarily due to macrophages [7]. Interestingly, CTFV treatment markedly reduced the levels of NO, iNOS, TNF-α, IL-6, and MCP-1 in the co-culture medium, indicating that the inflammatory process, or the interaction between macrophages and adipocytes, was attenuated by CTFV.

However, this study has certain drawbacks. The first drawback is that the form and efficacy of polyphenols and organic acids from CTFV may be different from those derived from the metabolic processes of animals and humans. Although the actions of polyphenols and organic acids, which are the main components of CTFV, are expected to contribute primarily to the anti-inflammatory efficacy, future experiments require comparative investigation of metabolites and safety analysis after CTFV treatment. In addition, the practical in vitro and in vivo applicability of polyphenolic substances isolated from CTFV is very low. Although polyphenolics undergo metabolism in liver and intestinal tissues by colon microbiota, most phytochemicals are not easily absorbed by the transcellular pathway because of size restriction (<600 Da) [52]. It has been reported that apple polyphenolics indirectly affect this pathway of cell signaling transduction by increasing the tight junction functionality [53], whereas polyphenol aglycones and glucosides are assumed to be taken up by sodium-glucose linked transporter (SGLT)-1 activation and passive diffusion [54,55]. After oral administration, certain polyphenols are absorbed poorly and can be excreted quickly, while others are absorbed rapidly by the intestinal barrier and reach the plasma in their original form. Consequently, the polyphenols reaching the target tissues and the blood may differ from their original forms due to the actions of the gastrointestinal tract, colonic microflora, and Phase I or Phase II reactions in the liver [56]. Therefore, understanding the metabolism, absorption, and bioavailability of polyphenols is important to elucidate their mechanisms or identify the final metabolites. The second drawback is concerning the reduction in stability of CTFV treated cells under neutral and culture conditions (pH 7 and 37 °C). Several studies have reported that polyphenolic substances in the cell culture medium are significantly degraded within 3 h in neutral buffer at 37 °C [57,58,59,60]. In particular, the concentrations of catechins and myricetin are significantly reduced within 10 min in DMEM at 37 °C in 5% CO_2_. Under the same conditions, quercetin, kaempferol, myricitrin, galangin, and flavonols are degraded within 3 h. It was reported that the concentrations of catechins were reduced by up to 30% when incubated for 24 h under neutral conditions. Therefore, the cell culture conditions (37 °C, pH 7, and treatment time of 24 h) in this study can affect the degradation and auto-oxidation of polyphenols in CTFV. Further optimization of the processing time and incubation conditions is required to elucidate the efficacy and stability of CTFV.

In conclusion, the present study revealed that CTFV produced from *C. tricuspidata* contains several bioactive ingredients with anti-inflammatory effects, and inhibits inflammation stimulated by the interaction of RAW264.7 and 3T3-L1 cells via suppressing the inflammatory biomarkers in a co-culture system. These findings could provide the basis for an effective strategy to alleviate inflammation-related disorders and develop functional food or dietary supplements.

## Figures and Tables

**Table 1:** The polyphenolic ingredients of CTFV.

Concentration of Polyphenolic Compounds (µg/g dw)					
	Chlorogenic Acid	Caffeic Acid	Rutin	Total Flavonoid (mg/mL)	Total Phenol (mg/mL)
CTFV	454.1 ± 5.2	60.8 ± 0.4	40.1 ± 2.9	0.12 ± 0.01	4.3 ± 0.2

Values are mean ± SD of 3 observations. Total phenol content is expressed in mg GAE/g dw. Total flavonoid content is expressed in mg QUE/g dw. CTFV, *C. tricuspidata* fruits vinegar.

**Table 2:** The parishin derivatives of CTFV.

Concentration of the Parishin Derivatives (µg/g dw)			
CTFV	Gastrodin	*p*-Hydroxybenzyl alcohol	Parishin A
	273.1 ± 4.6	182.9 ± 0.9	41.7 ± 0.3

Values are mean ± SD of 3 observations.
